# Problems and possibilities of studying malting quality in barley using molecular genetic approaches 

**DOI:** 10.18699/VJ21.021

**Published:** 2021-03

**Authors:** N.V. Trubacheeva, L.A. Pershina

**Affiliations:** Institute of Cytology and Genetics of the Siberian Branch of the Russian Academy of Sciences, Kurchatov Genomics Center of ICG SB RAS, Novosibirsk, Russia; Institute of Cytology and Genetics of the Siberian Branch of the Russian Academy of Sciences, Kurchatov Genomics Center of ICG SB RAS, Novosibirsk, Russia

**Keywords:** Hordeum vulgare, malting barley, QTL, marker-assisted selection, genome-wide association studies, Hordeum vulgare, пивоваренный ячмень, QTL, маркер-опосредованная селекция, полногеномный поиск ассоциаций

## Abstract

About one-third of the world’s barley crop is used for malt production to meet the needs of the brewing industry. In this regard, the study of the genetic basis of malting quality traits and the breeding of malting barley varieties that are adaptive to their growing conditions are relevant throughout the world, particularly in the Russian Federation, where the cultivation and use of foreign malting varieties of barley prevails. The main parameters of malting quality (artificially germinated and dried barley grains) are malt extract, diastatic power, Kolbach index, viscosity, grain protein, wort β-glucan, free amino nitrogen, and soluble protein content. Most of these components are under the control of quantitative trait loci (QTLs) and are affected by environmental conditions, which complicates their study and precise localization. In addition, the phenotypic assessment of malting quality traits requires elaborate, expensive phenotypic analyses. Currently, there are more than 200 QTLs associated with malting parameters, which were identified using biparental mapping populations. Molecular markers are widely used both for mapping QTL loci responsible for malting quality traits and for performing marker-assisted selection (MAS), which, in combination with conventional breeding, makes it possible to create effective strategies aimed at accelerating the process of obtaining new promising genotypes. Nevertheless, the MAS of malting quality traits faces a series of difficulties, such as the low accuracy of localization of QTLs, their ineffectiveness when transferred to another genetic background, and linkage with undesirable traits, which makes it necessary to validate QTLs and the molecular markers linked to them. This review presents the results of studies that used MAS to improve the malting quality of barley, and it also considers studies that searched for associations between genotype and phenotype, carried out using GWAS (genome-wide association study) approaches based on the latest achievements of high-throughput genotyping (diversity array technology (DArT) and single-nucleotide polymorphism markers (SNPs)).

## Abstract (ru)

Около одной трети урожая выращиваемого в мире ячменя используется для переработки в солод для обеспечения нужд пивоваренной промышленности. В связи с этим изучение генетической основы пивоваренных признаков и селекция пивоваренных сортов ячменя, адаптивных к условиям их произрастания, актуальны как во всем мире, так и в Российской Федерации, где преобладают выращивание и использование зарубежных солодовых сортов ячменя. К основным параметрам качества солода (искусственно пророщенного и высушенного зерна ячменя) относятся: экстрактивность, диастатическая сила, индекс Кольбаха, вязкость, содержание в зерне белка, β-глюкана, свободного аминного азота и растворимого белка. Большинство этих компонентов находится под контролем локусов количественных признаков (quantitative trait loci, QTL) и подвержено влиянию условий среды, что осложняет их изучение и точную локализацию. Кроме того, фенотипическая оценка пивоваренных признаков – трудоемкий и дорогостоящий процесс. В настоящее время известно более 200 QTL, связанных с пивоваренными параметрами, выявленных с привлечением двуродительских картирующих популяций. Молекулярные маркеры широко применяются как для картирования QTL-локусов, ответственных за пивоваренные качества, так и для выполнения работ по маркер-опосредованной селекции (МОС), что в комбинации с традиционными селекционными подходами дает возможность создавать эффективные стратегии, направленные на ускорение процесса получения новых перспективных генотипов. Тем не менее МОС пивоваренных признаков сталкивается с рядом трудностей, таких как невысокая точность локализации QTL-локусов, их неэффективность при переносе в другую генотипическую среду, сцепленность с нежелательными признаками, что обуславливает необходимость валидации QTL и сцепленных с ними молекулярных маркеров. В обзоре приведены результаты работ по использованию МОС для улучшения пивоваренных качеств ячменя, а также рассматриваются исследования по поиску ассоциаций между генотипом и фенотипом, выполненные с помощью ПГАА-анализа (полногеномный поиск ассоциаций) на основе последних достижений в области высокопроизводительного генотипирования (diversity array technology, DArT и single-nucleotide polymorphism, SNP маркеры).

## Introduction

Barley (Hordeum vulgare L.) ranks fourth in worldwide production, after wheat, rice, and maize. It is used for feed, food, and malting. About 30 % of the barley is processed into malt, mainly used for brewing beer (Newton et al., 2011; Bond et al., 2015; http://www.fao.org/faostat/ru/).

The amount and composition of the ingredients formed during malting (low-molecular-weight sugars, amino acids, fatty acids, and enzymes) affect the quality of malt (Bamforth, 2009). The quality of malt is mainly determined by the optimal values of malt extract (the amount of dissolved substances that pass into the solution during mashing, determined by measuring its relative density), diastatic power (the ability of enzymes to hydrolyze starch to simple sugars), viscosity (the solubility and filtration speed of the malt wort), content of β-glucan in the wort, Kolbach index (the solubility of malt protein), and contents of free amino nitrogen, soluble protein, and protein in the grain (Meledina et al., 2013; Cu et al., 2016). At the same time, it is necessary that the grain is of a suitable malting variety, has a high germinating capacity and energy, is sensitive to water absorption, does not have impurities, and does not contain microbial or chemical pollutants (Stanca et al., 2016).

In general, the main breeding goal is the development of barley varieties with high malting quality and increased yield (Li et al., 2009; Nikolaev et al., 2017). Maintaining a balance between these two parameters is a serious problem, since high yields, often dependent on the use of nitrogen fertilizers, are associated with high protein and β-glucan content, which is undesirable for high-quality malt (Chen et al., 2006). From a breeding point of view, there is no single barley ideotype universally accepted as describing a malting variety. Earlier, it was reported that two-row barleys were used for malting all over the world, except in the United States of America (USA) and Mexico, where six-row varieties were mainly used (Riggs, Kirby, 1978). The best malting varieties have a spring growth habit. However, due to the depletion of their genetic diversity (Laidò et al., 2009; Meledina et al., 2013), as well as climate change, interest in winter varieties has increased, and the brewing associations of Europe and the USA have included them in the list of recommended malting varieties (http://www.ukmalt.com/press-release-update-november-2019; https://ambainc.org/amba-publications/recommended-maltingbarley-varieties/).

The Russian Federation ranks first in worldwide production of barley and in areas featuring this crop (Varietal resources…, 2010; http://www.fao.org/faostat/ru/). However, breeding of malting barley in Russia has not been particularly developed; as a result, 80–90 % of malt is produced from imported raw materials or when growing foreign malting varieties (Goncharov, Mordovin, 2019). The breeding and cultivation of Russian malting barley varieties in the Russian Federation is carried out both in the European region and in Western Siberia and Altai (Surin et al., 2014; Aniskov et al., 2016; Nikolaev et al., 2017; Musalitin et al., 2019). Due to strict standards of the brewing industry and different climatic conditions in the various regions of the Russian Federation, the development of a raw material base for malting barley faces significant difficulties (Surin et al., 2014; Musalitin et al., 2019). As a rule, foreign varieties have good technological characteristics that meet the requirements of brewing production; however, when grown in Russian regions, the parameters of malt and beer produced from them often do not reach the declared characteristics (Aniskov et al., 2016; Nikolaev et al., 2017). In this regard, the development of competitive local varieties of malting barley that combine adaptability to growing conditions with optimal technological parameters is an important goal.

Malting quality traits belong to complex quantitative traits and have polygenic control (Fox et al., 2003), which makes it difficult to study them using conventional methods of analysis. The use of molecular markers makes it possible to significantly expand the possibilities for chromosomal localization of genes and QTLs (quantitative trait loci) that determine malt quality characteristics and provide breeders with an effective tool for accelerated and directed plant selection (marker-assisted selection) (Han et al., 1997). 

This review examines and discusses the main problems associated with molecular genetic mapping of malting quality traits, as well as the results of using recent high-throughput genotyping technologies for applied research to obtain breeding material with improved malting characteristics.

## Genetic control of barley malting characteristics

The phenotype that determines the malting quality of barley is the result of interactions among a large number of components, each of which shows a complex inheritance (Molina-Cano et al., 1997; Fox et al., 2003). Most of them are quantitative traits with a comparatively low heritability, which are controlled by multiple genes (Fox et al., 2003). For example, the mean heritability for malt extract assessed in the F2 and F3 generations using different methods and populations ranged from 8 to 70 %, whereas the heritability of α-amylase activity in F2 and F5 plants ranged from 37 to 65% and from 39 to 74 %, respectively (Foster et al., 1967). In addition, the phenotypic variation of quantitative traits often depends on growing conditions, such as soil composition, temperature, irrigation, fertilizer application (Qi et al., 2005), genotype×environment interactions (Coles et al., 1991), methods of laboratory analysis (Cullis et al., 2003), and complex relationships among components that determine malting quality. All these aspects make it difficult to accurately localize the QTLs that control malting quality traits.

In some studies, QTLs for certain malting quality traits were found in different regions of the genome, due to the influence of different genotypes used in cross populations and/or the influence of genotype×environment interactions. For example, QTLs controlling the content of malt extract were identified on chromosomes 1H and 2H in populations derived from two North American varieties (Marquez-Cedillo et al., 2000) and on chromosomes 1H and 5H in populations from Australian and Canadian varieties (Collins et al., 2003). Even when using the same population (Blenheim×E224/3), QTLs for malt extract on chromosome 2H were found by different researchers in different amounts and positions (Thomas et al., 1996; Powell et al., 1997). This makes it necessary to validate QTLs using different mapping populations grown under different conditions in order to assess their interaction with the environment (Panozzo et al., 2007; Elía et al., 2010). 

However, QTL analysis based on biparental mapping populations has been widely used to identify and localize QTLs (Marquez-Cedillo et al., 2000; Collins et al., 2003; Edney, Mather, 2004; Emebiri et al., 2005; Panozzo et al., 2007; Rae et al., 2007). QTLs or genes controlling malting traits were identified on all seven barley chromosomes, but most were identified on chromosomes 1H, 4H, 5H, and 7H (Schmalenbach, Pillen, 2009; Wang et al., 2015). Many studies investigating QTLs related to malting quality were based on genotyping data obtained using various molecular markers (Han et al., 1997; Mather et al., 1997; Coventry et al., 2003; Panozzo et al., 2007; Rae et al., 2007; Schmalenbach, Pillen, 2009; Szűcs et al., 2009; Castro et al., 2013). In addition, the database of barley markers has significantly expanded with the development of methods for detecting SNPs (single-nucleotide polymorphisms) using Illumina GoldenGate technology, which provided access to thousands of alleles and led to the creation of a high-density consensus genetic map of barley containing 2943 SNP loci (Close et al., 2009). Information about these SNPs was combined with other genetic markers such as RFLP, AFLP, SSR, and diversity array technology (DArT) in the integrated barley malting QTL database (Szűcs et al., 2009). As a result, a map was compiled with 154 QTLs associated with 18 malting quality traits localized on all barley chromosomes.

At least 268 malting QTLs/genes are known to have been identified in more than 20 mapping populations (Hayes et al., 2000; Zale et al., 2000; Fang et al., 2019). However, the results of these studies are difficult to directly apply to breeding for many reasons. For example, most mapping populations do not include genotypes used to produce new varieties, QTLs may be specific to a particular population, alleles desirable for malting quality may only be fixed in certain genotypes, and some QTLs may have low localization accuracy due to the small size of mapping populations (Sneller et al., 2009). A particular problem for breeding is that QTLs identified in mapping populations may not segregate in breeding populations, such as QTLs for malting quality traits on barley chromosomes 4H and 7H (Condon et al., 2008). In this regard, it is emphasized that the use of local breeding lines for mapping can be more effective in identifying QTLs that are adequate to specific growing conditions and breeding goals (Pozniak et al., 2012).

## Using marker-assisted selection to improve malting qualities

The marker-assisted selection (MAS) of barley is of particular interest in terms of developing genotypes with good malting quality, since the phenotypic evaluation of malting quality characteristics using laboratory equipment is an expensive process and requires large amounts of grain. In addition, these traits are affected by the interaction of the genotype with the environment. Molecular markers for assessing malting quality traits can provide rapid selection of plants at the early stages of breeding through a study of large populations, thereby increasing the likelihood of detecting the desired genetic combinations (Igartua et al., 2000).

Marker-assisted selection for quantitative traits, which include malting quality traits, has two main limitations. First, in comparison with monogenic traits, quantitative traits are characterized by low heritability, which leads to a less accurate assessment of their genetic localization. As a result, it is necessary to select a large fragment of the chromosome, which is associated with the transfer of many potentially undesirable genes. Second, many of the QTL alleles are difficult to detect when transferred to a different genetic background (Rae et al., 2007). Most studies on QTL mapping for malting quality traits were based on crosses of parents contrasting in malting traits, for example, malting variety×feed variety, which goes against the common breeding practice for malting barley, whereby feed genotypes are not typically used. In this regard, QTLs for malting quality traits should be verified in breeding programs before being used in marker-assisted selection. In addition, some of the identified QTLs cannot be used in MAS since they are associated not only with target traits, but also with undesirable ones. For example, one of these QTLs found on the long arm of chromosome 3H was associated not only with an increase in diastatic power, but also with an increase in viscosity (Panozzo et al., 2007).

One successful example of improving malting quality using MAS is the work related to the enzyme β-amylase, which mainly determines the diastatic power (Zhang et al., 2007). The Bmyl locus on chromosome 4H controls β-amylase activity, free/bound enzyme ratio, and thermal stability, and its alleles are different isoenzyme types. PCR markers have been developed that allow selection of different alleles of β-amylase, which allows the use of these markers in MAS depending on the needs of the brewing industry (Erkkilä, 1999). For example, if high diastatic power and enzymatic activity are required, the Sd2-H and Sd3 alleles should be selected. Using molecular markers and double-haploid technology, the Bmy1-Sd3 allele from Hordeum spontaneum L. was transferred to two commercial barley varieties. As a result, the activity of β-amylase and, thus, the diastatic power in these varieties increased by an average of 30 % (Li et al., 2004). The use of the CAPS marker made it possible to transfer the Sd3 allele of the thermostable β-amylase from wild barley (H. spontaneum) into a commercial barley (variety Gairdner) and obtain elite lines with high malting quality characteristics (Xu et al., 2018). Supplementary material^1^ presents a description of the markers used in MAS for barley malting characteristics.

Supplementary Materials are available in the online version of the paper: https://vavilov.elpub.ru/jour/manager/files/SupplTrubacheeva_Engl.pdf


Cultivated barley contains two isoforms of the enzyme lipoxygenase, which oxidizes unsaturated fatty acids to the corresponding hydroxyperoxides. One of the isoforms, LOX1, promotes the synthesis of substances that impair the flavor stability of beer (Hirota et al., 2006). It was found that this trait is encoded by a locus on chromosome 4H, and the absence of this protein is caused by a single-nucleotide mutation. The use of the CAPS marker for the selection of mutants lacking this protein made it possible to develop new breeding lines in three years, despite this process usually taking approximately ten years. Beers made with barley lacking LOX1 (null-Lox variety) were found to have a 75 % reduction in the content of substances that cause a stale flavor due to oxidation compared to beer made from ordinary barley malt (Hirota et al., 2005). One of the indicators of beer quality is the stability of beer foam, which depends on the combined action of various proteins, iso-alpha acids, polysaccharides, and metal ions contained in beer. To select haplotypes of Z4 and Z7 proteins associated with the quality of beer foam, CAPS markers were developed, and their efficiency was shown in the analysis of 23 malting barley varieties (Iimure et al., 2009).

The possibility of using MAS to select populations with improved malting quality has been shown (Coventry, et al., 2003). For example, it was found that lines carrying an allele linked to SSR marker EBmac501 on chromosome 1H were characterized by increased diastatic power, as well as β-amylase and α-amylase activities, compared to other lines. In addition, this marker locus was associated with an increased content of malt extract and, therefore, was considered promising for use in MAS (Collins et al., 2003). The use of MAS for the selection of plants carrying the target malting quality traits made it possible to develop promising breeding lines when crossing feed barley Keel with three donor varieties with high malting quality characteristics (Vassos et al., 2004). F. Han et al. (1997) compared the efficiency of malting quality trait selection using phenotypic assessment and marker-assisted selection using molecular markers flanking the QTL1 and QTL2 genome regions for malt extract, α-amylase activity, diastatic power, and β-glucan content. It was shown that, for QTL1, the combination of MAS and phenotype assessment was more effective than phenotypic selection only, which involves laborious and expensive procedures. The selection of desired genotypes can be greatly facilitated using PCR markers; therefore, a number of RFLP markers for malting QTLs have been converted to PCR markers (Lee, Penner, 1997).

A number of other examples of the use of MAS to identify QTLs associated with malting quality traits are also known. For example, the localization of two QTLs affecting malting quality traits on chromosome 5H was confirmed using molecular markers. Later, the selection of genotypes carrying alleles from the malting variety Harrington made it possible to obtain double-haploid lines with improved malting characteristics, such as low β-glucan values and protein content in grain, high diastatic power, and high malt extract (Igartua et al., 2000). The use of PCR markers for the QTL region on chromosome 5H affecting α-amylase activity made it possible to introgress this trait from the malting barley variety Morex to the feed barley Labelle (Ayoub et al., 2003). In addition, this study showed that MAS can be successfully applied to incorporate QTLs into populations where only one of the parents (the Morex variety) was used for the initial QTL identification and mapping. Using SSR markers, the QTL regions for protein content, malt extract, and viscosity were introgressed from the winter malting barley Nure to the double-haploid population obtained from crossing Nure with the spring malting variety Tremois (Laidò et al., 2009). SSR markers flanking QTLs on chromosomes 2H, 6H, and 7H were developed. These loci had a significant effect on protein content and, according to the authors, may be useful in the development of varieties with a high protein content (Fan et al., 2017). Using the populations obtained by crossing elite malting barley varieties, QTLs for malting quality traits were mapped, and two SSR markers promising for use in MAS were identified (Panozzo et al., 2007). 

## Genome-wide association analysis as a perspective for the development of molecular selection of malting quality traits

The emergence of more cost-effective, high-throughput genotyping platforms such as diversity array technology (DArT) (Wenzl et al., 2004) and Illumina’s GoldenGate assay (Close et al., 2009), as well as improvements in statistical methodology and computer programs, has enabled genome-wide association studies (GWAS) to be a promising alternative approach to conventional QTL analysis of biparental populations for the detection and accurate mapping of quantitative trait loci. The advantages of this method include a wider coverage of the genetic diversity of the population, i. e., simultaneous study of a large number of alleles, high-resolution mapping, establishment of single-nucleotide polymorphisms, and reduced study time due to the absence of the need to develop a mapping population (Rafalski, 2010). 

For the effective use of available technologies, a number of researchers used information obtained during long-term breeding trials when performing GWAS, which significantly reduced the cost of genetic research. For example, data on malting quality traits from 97 breeding trials conducted on 1862 lines were combined with the results of using 3072 SNP markers for association mapping. This approach was found to provide improved accuracy for identifying QTLs associated with malting quality traits compared to previous mapping studies (Mohammadi et al., 2014). In addition, the GWAS method can identify a much larger number of molecular markers compared to traditional QTL mapping (Cai et al., 2013). In another study, phenotypic data of 18 malting traits accumulated over 25 years for 174 European barley varieties were used in the study of GWAS using DArT markers. 

In addition to confirming the already known QTLs on chromosomes 1H, 2H, and 5H, new associations were found, for example, markers linked to the malting quality and viscosity (Matthies et al., 2014). A collection of 91 elite malting barley lines was analyzed using association mapping to identify DArT markers associated with seven malting traits, and 19 putative candidate expressed sequence tags responsible for marker-trait associations were identified (Beattie et al., 2010). The study of a collection of 224 spring barleys using 1536 SNPs made it possible to detect 57 novel QTLs responsible for agronomic valuable traits, including starch and protein content (Pasam et al., 2012). Thus, the associations between genotype and phenotype identified in the studies reviewed may be useful for selecting parent genotypes carrying the desired alleles in order to model future breeding studies, although the results obtained need to be validated in the field.

## Conclusion

The malting quality of barley is the result of a complex interaction of various components controlled by multiple genes. In this regard, selection based on phenotypic characteristics is a time-consuming and expensive process. Marker-associated selection of malting quality traits is an effective alternative or complement to conventional breeding, but requires detailed information about genes/QTLs responsible for the target traits. QTL analysis is widely used for the chromosomal localization of agronomic traits and the detection of molecular markers. To date, a large number of QTLs have been identified that control malting quality traits, and the use of associated molecular markers and recent advances in molecular tools for high-resolution genotyping make it possible to effectively select the desired genotypes for breeding barley varieties with high malting quality characteristics.

## Conflict of interest

The authors declare no conflict of interest.
